# When diagnostics outpace decisions: mimicry and expansibility in tropical infectious diseases

**DOI:** 10.1186/s41182-025-00847-w

**Published:** 2025-11-28

**Authors:** Hidenori Takahashi

**Affiliations:** Department of Respiratory Medicine, Tokyo Shinagawa Hospital, Tokyo, Japan

## Abstract

Diagnostic advances outpace bedside interpretation in tropical medicine. Two forces drive this gap: mimicry—infectious syndromes resembling noninfectious disease, and expansibility—epidemiology transcending geography, age, season, and host. The result is misclassification, mistargeted therapies (e.g., steroid-treated helminth infection), and wasted resources amid climate- and mobility-driven shifts. This correspondence proposes lightweight, locally led evidence circulation through structured case reviews, minimal essential data, and living, site-specific algorithms that integrate mimicry-aware red flags and calibrated pretest probabilities. Such networks transform tacit experience into auditable knowledge, improve day-to-day decision-making, and align technological advances with context, thereby strengthening equitable and sustainable care for tropical diseases.

In the twenty-first century, tropical medicine faces a widening gap between diagnostic advances and the local knowledge needed to translate results into appropriate treatment (Fleming et al. in Lancet 398:1997–2050, 2021; Albarqouni et al. in BMJ Glob Health 7, 2022). Imaging, immunological assays, and molecular detection are now available in many low- and middle-income countries, enabling the identification of pathogens that were previously difficult to detect (Fleming et al. in Lancet 398:1997–2050, 2021). Yet, the bedside loop from “detection → diagnosis → treatment” remains fragile: clinical decision-making and the sharing of case-based knowledge have not kept pace with technological progress (Albarqouni et al. in BMJ Glob Health 7, 2022).

Two intersecting properties underlie this gap: (i) mimicry—tropical infections presenting as other, unrelated diseases, and (ii) expansibility—epidemiology breaking traditional boundaries of geography, age, season, and host background. Even in an era of portable diagnostic devices, if the initial clinical premise is wrong, technology does not improve outcomes or resource allocation (Albarqouni et al. in BMJ Glob Health 7, 2022).

## Mimicry

Tropical infections frequently masquerade as other conditions, complicating recognition. *Plasmodium falciparum* malaria can present with features resembling hematologic disease [2]. Pulmonary paragonimiasis and schistosomiasis may mimic autoimmune disorders and sometimes result in inappropriate steroid therapy. A recent case of pulmonary paragonimiasis closely mirrored IgG4-related disease and even showed a transient steroid response before the true infection was identified [3]. When variability in radiologic or histopathologic interpretation coincides with limited access to confirmatory molecular tests, both unnecessary treatment and missed diagnoses can occur, harming patients and misallocating scarce resources [1]. Overdiagnosis of malaria has been documented in sub-Saharan Africa and is associated with substantial annual losses, reaching tens of millions of USD, alongside failures to treat alternative causes of fever [2, 4]. Thus, mimicry fosters not only individual diagnostic errors but also systematic distortion [2].

## Expansibility

Population aging, increasing comorbidity, greater travel and migration, and climate-driven shifts in vector ecology are redefining the epidemiology of tropical infections [5]. In temperate regions, West Nile virus illustrates this age gradient: introduced to North America in 1999 and now established across much of the continent, it causes seasonal neuroinvasive disease with disproportionately high morbidity and mortality in older adults [6].

Dengue shows the same pattern at the age extremes—including older adults—and severity is driven chiefly by antibody-dependent enhancement rather than thrombocytopenia; it is, therefore, no longer confined to a “childhood disease” [5]. Geographically, arboviruses have extended beyond their historical ranges: southern Europe has recorded autochthonous dengue since 2010, and Japan experienced a domestic outbreak in 2014 after nearly 70 years, with imported cases continuing thereafter [5].

Chikungunya likewise expanded across tropical and subtropical regions during 2025, with outbreaks documented in the Indian Ocean, parts of Africa, and South Asia, and with increases reported in the Americas [7]. Expansibility renders clinicians’ tacit priors—based on region, age, and season—obsolete, increasing the risk of misinterpreting identical test results and misweighting clinical decisions [5].

At the same time, knowledge production remains imbalanced. Due to historical and systemic factors, initial publications on tropical infections often originate from urban tertiary centers in high-income countries rather than from endemic, lower-income settings [8]. As a result, evidence and guidelines may not fully reflect local pretest probabilities or region-specific red flags, and feedback from endemic frontlines is weakened [8].

To address these gaps, a multi-level approach is needed. At the meso-level, lightweight, locally led evidence-circulation networks—regular case reviews using a standardized template and minimal essential data—can support day-to-day decisions, linking facility → region → multi-center → international without over-centralization. Where communication is limited, remote mentoring in a hub-and-spoke design (Project ECHO-type) can horizontally disseminate case knowledge and connect peripheral facilities with central expertise [9]. Case accumulation is a means; the purpose is to improve decision-making and build a reproducible, practice-rooted knowledge base. Networks maintain living algorithms with site-specific pretest probabilities and mimicry-aware red flags, updated in small, logged cycles. This keeps bedside interpretation calibrated and regulates high-stakes therapies. Most importantly, locally generated diagnostic acumen can surface misclassification and evidence biases and feed back into meta-analyses and guideline updates beyond the originating settings [2, 4, 8].

At the macro-level, long-term collaborative research and capacity-building, such as the sustained partnerships formed around avian influenza H5N1, have strengthened clinical understanding and surveillance over time [10]. International cooperation remains a cornerstone for steadily reinforcing diagnostic and therapeutic infrastructure in endemic regions.

These levels are complementary. The value of technology depends on robust decision frameworks and the open sharing of results. Converting scattered clinical experience into durable, accessible knowledge—and circulating it widely—aligns with the principle of equitable data sharing emphasized in discussions on a WHO pandemic agreement, and it reinforces the foundations of regional surveillance. Ultimately, sustainable global health for tropical diseases rests on two pillars: locally cultivated clinical wisdom and widely disseminated scientific evidence.

## Data Availability

No data sets were generated or analysed during the current study.
